# Identification of expression patterns in the progression of disease stages by integration of transcriptomic data

**DOI:** 10.1186/s12859-016-1290-4

**Published:** 2016-11-22

**Authors:** Sara Aibar, Maria Abaigar, Francisco Jose Campos-Laborie, Jose Manuel Sánchez-Santos, Jesus M. Hernandez-Rivas, Javier De Las Rivas

**Affiliations:** 1 Bioinformatics and Functional Genomics research group, Cancer Research Center (IMBCC, CSIC/USAL) and Instituto de Investigación Biomédica de Salamanca (IBSAL), Salamanca, Spain; 2 Unidad de Diagnóstico Molecular y Celular del Cáncer, Cancer Research Center (IMBCC, CSIC/USAL) and Instituto de Investigación Biomédica de Salamanca (IBSAL), Salamanca, Spain; 3 Servicio de Hematología, Hospital Universitario de Salamanca (HUS/IBSAL/USAL), Salamanca, Spain; 4 Department of Statistics, University of Salamanca (USAL), Salamanca, Spain

**Keywords:** Disease stage, Disease subtype, Disease progression, Cancer, Leukemia, Transcriptomics, Gene expression, Gene signature, Expression profiling, Expression pattern, Bioinformatics, Pattern recognition, Data integration

## Abstract

**Background:**

In the study of complex diseases using genome-wide expression data from clinical samples, a difficult case is the identification and mapping of the gene signatures associated to the stages that occur in the progression of a disease. The stages usually correspond to different subtypes or classes of the disease, and the difficulty to identify them often comes from patient heterogeneity and sample variability that can hide the biomedical relevant changes that characterize each stage, making standard differential analysis inadequate or inefficient.

**Results:**

We propose a methodology to study diseases or disease stages ordered in a sequential manner (e.g. from early stages with good prognosis to more acute or serious stages associated to poor prognosis). The methodology is applied to diseases that have been studied obtaining genome-wide expression profiling of cohorts of patients at different stages. The approach allows searching for consistent expression patterns along the progression of the disease through two major steps: (i) identifying genes with increasing or decreasing trends in the progression of the disease; (ii) clustering the increasing/decreasing gene expression patterns using an unsupervised approach to reveal whether there are consistent patterns and find genes altered at specific disease stages. The first step is carried out using *Gamma rank correlation* to identify genes whose expression correlates with a categorical variable that represents the stages of the disease. The second step is done using a *Self Organizing Map* (*SOM*) to cluster the genes according to their progressive profiles and identify specific patterns. Both steps are done after normalization of the genomic data to allow the integration of multiple independent datasets. In order to validate the results and evaluate their consistency and biological relevance, the methodology is applied to datasets of three different diseases: myelodysplastic syndrome, colorectal cancer and Alzheimer’s disease. A software script written in R, named *genediseasePatterns*, is provided to allow the use and application of the methodology.

**Conclusion:**

The method presented allows the analysis of the progression of complex and heterogeneous diseases that can be divided in pathological stages. It identifies gene groups whose expression patterns change along the advance of the disease, and it can be applied to different types of genomic data studying cohorts of patients in different states.

**Electronic supplementary material:**

The online version of this article (doi:10.1186/s12859-016-1290-4) contains supplementary material, which is available to authorized users.

## Background

Despite the enormous amount of omic datasets produced from recent biomedical clinical studies of complex diseases –like cancer or neurodegenerative disorders–, the integration and efficient analysis of these types of large scale data to achieve a better characterization of the stages of each disease still remains a challenge. In fact, there are genome-wide expression studies from clinical samples of complex pathologies that present subtypes of the disease in a progressive way, evolving from low-risk and good prognostic stages to high-risk and poor prognostic stages. The correct analysis of these stages is very relevant to find the genes that mark the phases and progression of a disease and it can not be done by standard differential expression analyses. The algorithms to analyze time-series can allow the search for progressive changes in the genes along several conditions but these methods need the time as a key parameter to be run [1, 2]. However, many biomedical studies have to analyze patients in different clinical stages of the disease without a clear temporal relation.

The patient heterogeneity present in samples from clinical cohorts can hide biomedical relevant changes associated to the progression and prognosis, making the standard pairwise comparisons between diseases subtypes inadequate or inefficient. Moreover, the studies of disease subtypes often demands increasing the size of the patient cohorts collecting datasets coming from different hospitals or research sources that, frequently, have been obtained with different platforms or in different batches. This renders the integration problem even harder. To overcome these limitations, we developed a methodology which allows studying the gene expression transcriptomic profiles of related disease subtypes using an approach that is robust to signal variability. Rather than using differential expression analysis to look for specific markers for each subtype, our approach is based on a non-parametric co-expression profiling along the different stages of the disease followed by the application of a pattern recognition method. This allows unravelling similarities and identify specific gene patterns associated to the stages or progression of the disease. The procedure was initially designed for the analysis of *myelodysplastic syndromes* (MDS), which constitute a heterogeneous group of hematological diseases which often evolve to acute leukemia. However, the method is generalized to be applicable to the study of other diseases with stages, and here we illustrate its application to two other experimental cohorts of patients from *Alzheimer’s disease* (AD) and from *colorectal cancer* (CRC), where a clear clinical characterization of the individuals in stages has been done. All these datasets have been produced with high-density microarray expression platforms; therefore, as a validation, we also applied the methodology to a simulated RNA-seq dataset where a subset of genes have been modeled to follow progressive changes in several stages.

## Methods

### Experimental datasets including categorized samples of well-defined diseases

The first dataset analyzed in this study corresponds to bone marrow samples (bone marrow mono-nucleated cells) from a cohort o*f myelodysplastic syndrome (MDS*) patients of four subtypes, plus a subset controls that did not have the disease (i.e. healthy bone marrows) and a subset of samples from patients with acute myeloid leukemia (AML, the severe malignant disease where many high-risk MDSs progress to) (Table 1). The MDS data were taken from GEO (GSE 13159, https://www.ncbi.nlm.nih.gov/geo/query/acc.cgi?acc=GSE13159) [3], that includes a large genome-wide expression study of patients with different types and subtypes of leukemia with 2,096 samples (i.e. many different hematological malignancies) done by an international consortium (MILE Study). Within this cohort, the subset of MDS patients was only of 206, and we selected 53 of them for our study. The World Health Organization (WHO), based on morphologic evaluation of bone marrow cells and genetic abnormalities, classifies MDS into 6 major subtypes: (i) refractory cytopenia with uni-lineage dysplasia (RCUD, that usually corresponds mainly to refractory anaemia); (ii) refractory anaemia with ring sideroblasts (RARS); (iii) refractory cytopenia with multi-lineage dysplasia (RCMD); (iv) refractory anaemia with excess blasts (RAEB-1, <5 % blasts); (v) refractory anaemia with excess blasts (RAEB-2, with 5–19 % blasts); (vi) MDS associated with isolated del(5q) (that gives a specific disease: 5q-syndrome) [4]. Blast percentage of more than 20 % defines the entrance into AML. As indicated above, in this study we selected only 53 MDS (coming from 2 Hospitals) because we needed MDS samples with clear clinical information, including the level of blast cells (i.e., known % of immature cells in the bone marrow of the studied patients), as well as MDS subtypes that can evolve to acute leukemia. For this reason, we excluded “MDS with ring sideroblasts” (RARS) that is a low-risk subtype with a very strong iron signature that usually does not evolve to AML, and “5q-syndrome” that defines a very specific MDS subtype. Moreover, only MDS samples with “normal karyotype” were selected, avoiding cytogenetic abnormalities because these alterations cause MDS subtypes that usually have distinct clinical characteristics and treatments. The platform used to measure expression in all these sample set was the *Affymetrix* Human Genome U133 Plus 2.0 Array, that includes readings for 18,950 human genes.

**Table 1:** Number of samples in each dataset of the studied diseases (myelodysplastic syndrome MDS, Alzheimer's disease AD and colorectal cancer CRC) divided in stages and ordered according to the progression of each disease

	Disease subtypes and number of patient samples					
Myelodysplastic Synd. (MDS)	Control	RCUD	RCMD	RAEB1	RAEB2	AML
Patient samples (N)	11	6	17	4	5	10
Alzheimer’s disease (AD)	Control	Incipient	Moderate	Severe		
Patient samples (N)	8	7	8	7		
Colorectal cancer (CRC)	Control	Stage 1	Stage 2	Stage 3	Stage 4	
Patient samples (N)	25	13	37	34	20	

MDS dataset includes 53 samples in 6 stages. AD dataset includes 30 samples and 4 stages. CRC dataset includes 129 samples and 5 stages. The controls correspond in all cases to samples of individuals without the disease. The stages are placed according to the progression of the diseases from the controls (no-disease) to the more acute or severe pathological states

The second dataset corresponds to samples from hippocampus from a cohort of *Alzheimer’s disease* (AD) patients diagnosed at three progressive states of the disease as classified by the neurologists: incipient, moderate and severe; plus some control samples of normal hippocampus from individuals without the disease (Table 1). The dataset including 30 samples was taken from GEO (GSE28146, https://www.ncbi.nlm.nih.gov/geo/query/acc.cgi?acc=GSE28146) [5] and the samples were generated using laser capture micro-dissection to selectively collect CA1 hippocampal gray matter. In this way, this clinical cohort –despite the small size– it is very well controlled and allows focusing the study on the gene expression alterations of one specific region of the brain that is most affected in AD [5]. The dataset was generated using the *Affymetrix* Human Genome U133 Plus 2.0 Array.

The third dataset corresponds to primary tumor samples from a cohort of 104 patients with *colorectal cancer* (CRC) categorized into four main stages of the tumor, plus 25 samples from homogenized normal tissue that were used as reference control colon samples (Table 1). Since this dataset included the tumor grade within the clinical information, we applied the methodology on the samples classified in 4 main tumor stages, without considering sub-stages which split the set into smaller groups but that do not correspond to distinct pathological stages as defined by the oncologists (also because such sub-stages would contain few samples). All these samples were taken from GEO (GSE21510, https://www.ncbi.nlm.nih.gov/geo/query/acc.cgi?acc=GSE21510) [6] that includes a total set of 129. The disease samples correspond to cancer cells in 104 patients with CRC isolated using laser micro-dissection to have optimum homogeneous cellular material and avoid contamination by non-tumoral cells. This dataset was also generated using the *Affymetrix* Human Genome U133 Plus 2.0 Array.

On the AD and CRC datasets the analyses were done using directly the processed matrix downloaded from GEO. These data matrices use the *Affymetrix* identifiers (i.e., probesets) for the genes, which were used in the whole process, and only at the end the probesets were mapped to genes, ignoring ambiguous probes [7]. The functional enrichment analyses done on the different gene lists identified in this work were performed using the bioinformatic tools *Enrichr* [8] and *GeneTerm Linker* [9].

### Definition of stages or pathological subtypes along the progression of each disease

The main requisite underlying the analytic methodology proposed is that the studied disease –i.e. MDS, AD, CRC– presents different progressive stages, ranging from early stages (e.g. lower malignancy or good prognosis) to late stages (e.g. advanced stages usually ligated to bad prognosis). In order to find genes as marking features whose expression levels (expression signal) can be associated to the level of malignancy, the algorithm searches for genes whose expression evolves following an increasing or decreasing trend along the disease stages: being lower at earlier stages and higher at later stages or vice versa. In this analysis it is important to keep the context of the disease progression, in addition to the samples grouped and sorted by disease stage. It is also recommended to have at least one reference stage. Typically, this will be a control or healthy stage taken as origin. To provide more statistical power, it is recommended to include a reference of the most malignant stage, as terminal, even when in some cases it might not be the focus of the study. The use of these reference stages, initial and terminal, is specially important when the intermediate stages may be heterogeneous, fuzzy or not very well defined from the pathological point of view. This is, for example, the case for MDSs where separation between different subtypes of low-risk MDS stages is frequently not well done or not easy to do, even for the hematologist doctors, because it can be easily confused with aplastic anemia [10].

Another point to take into account is the number of stages included in the study of a disease. More stages will provide more statistical power to calculate the correlation and find patterns. However, in some cases it might be interesting to analyze fewer steps in the progression of the disease in order to concentrate the number of samples and obtain a more consistent and repetitive result.

**Fig. 1:**
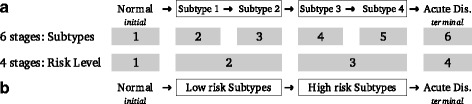
Example of disease stages. Scheme showing two ways of setting up the stages of a disease taking MDS case as example. In both cases the stages must be placed in progressive order considering one initial stage, usually taken as control or normal stage, and one terminal stage that usually corresponds to the most severe or acute stage of the disease (Acute Disease). The stages are considered as discrete –i.e. not as continuous variables– and independent –since they correspond to the evolution measured in different individuals–. **a** An example for 6 stages taken from the MDS case, considering different disease subtypes. **b** An example for 4 stages taken from the MDS case, considering only low-risk and high-risk subtypes

Considering all the described conditions the experimental datasets where divided in groups of samples according to stages of patients defined by the experts on each disease. In this way, the MDS (*myelodysplastic syndrome*) dataset analyzed in this study allowed two possible approaches: (a) applying the methodology considering 4 MDS disease subtypes (RCUD, RCMD, RAEB1 and RAEB2); or (b) grouping the subtypes in two levels by the risk of transforming into acute leukemia (i.e. RCUD and RCMD into low-risk MDSs, and RAEB1 and RAEB2 into high-risk MDSs). Therefore, when including the no-leukemia samples (NoL) as the initial control stage and AML as the terminal malignant stage, these resulted into two possible scenarios for analysis: (A) 6 stages contrast: NoL, RCUD, RCMD, RAEB1, RAEB2, AML; and (B) 4 stages contrast: NoL, Low-Risk MDS, High-Risk MDS, AML (Fig. 1). In the case of the AD (*Alzheimer’s disease*) dataset, 3 progressive states of the disease were considered: incipient, moderate and severe; plus the control samples corresponding to the normal hippocampus from individuals without the disease (Table 1). In the case of the CRC (*colorectal cancer*) dataset, 4 progressive tumor stages were considered: stage 1, stage 2, stage 3, stage 4; plus the controls corresponding to the normal colon tissue (Table 1).

### Simulated RNA-seq dataset including a subset of genes altered along several stages

To provide another validation of the method we performed an analysis on simulated data corresponding to a RNA-seq sample set of a model disease divided in 6 stages, where a small subset of genes have a pattern of up-regulation or down-regulation along the stages. The dataset for this simulation was produced with the R package *SimSeq* which includes a data-based algorithm to allow a non-parametric simulation of RNA-seq data [11]. The experimental dataset used as template to generate the simulated data was a RNA-seq data matrix including 20,531 genes and 72 samples in paired columns corresponding to individuals with Kidney Renal Clear Cell Carcinoma (36 replicates in 2 conditions: control and treated groups). Using this data we random-selected 1000 genes and 18 individuals of the same condition (control) to generate the simulated distributions. The individuals were divided in 6 groups of 3 replicates that correspond to 6 simulated disease stages. Then, 200 random-selected genes where modified in their expression to have a small change in each stage following 4 different trends: 50 genes up with larger changes at the initial stages; 50 genes up with larger changes at the late stages; 50 genes down with larger changes at the initial stages; 50 genes down with larger changes at the late stages. All the other 800 genes did not followed any trend along the stages, despite having different expression intensities and expression variabilities along the samples reflecting a typical RNA-seq expression distribution.

### *Gamma* rank correlation

To search for genes whose expression tends to increase or decrease as the disease progresses, we calculate the correlation between the expression of each gene and a variable that represents the level/stage of the disease (a categorical/ordinal variable). To do so, we use *Goodman and Kruskal’s Gamma* statistic [12], that is a rank-based correlation measure that calculates the number of inversions in rank ordering for two variables compared. This coefficient is specially recommended when there are many ties in any of the variables. In R, we calculate the *Gamma rank correlation* through the package *RoCoCo* (version 1.1.2) [13], that provides the implementation of several rank correlation measures taking into account some peculiarities for noisy data. In this way, *Gamma* is calculated as the subtraction of the number of concordant pairs (*C*, cases with the same order in both variables) minus the number of discordant pairs (*D*, cases with different order), divided by the total number of concordant and discordant pairs:

$$ \gamma =\frac{C-D}{C+D} $$

In most implementations, the ranking for the variables is constructed in a strict manner (i.e. 1.300000001 is considered bigger than 1.30). Since in case of noisy data, this can distort the results [14], the authors of the *RoCoCo* package included an additional parameter, named *r*, which determines the margin in which both values will be considered equal, and therefore tied in the ranking [15]. In all the runs included in this study, we used for *r* a 10 % of the interquartile range of the variables (i.e. the genes)*.* We also set to "linear" the family of similarities to compare the order of the variables (*R*), and "min" as t-norm (triangular function) to determine the aggregation of the ordering measures (*T*). Finally, to calculate the *p-value* we used the default value of 1000 permutations, which was enough to test whether the association was significant at 95 %, while keeping a reasonable execution time. The *p-values* were adjusted for multiple-testing using False Discovery Rate (FDR) [16] (implemented in *p.adjust* function in R) and considering the total number of genes present in the measuring platform (i.e. the microarray). The genes with a significant *Gamma* (i.e. absolute *Gamma* > 0.50, and FDR adjusted *p*-value < 0.05) were selected as significantly correlated with the stages and progression of the disease.

### Features profiling and patterns recognition

**Fig. 2:**
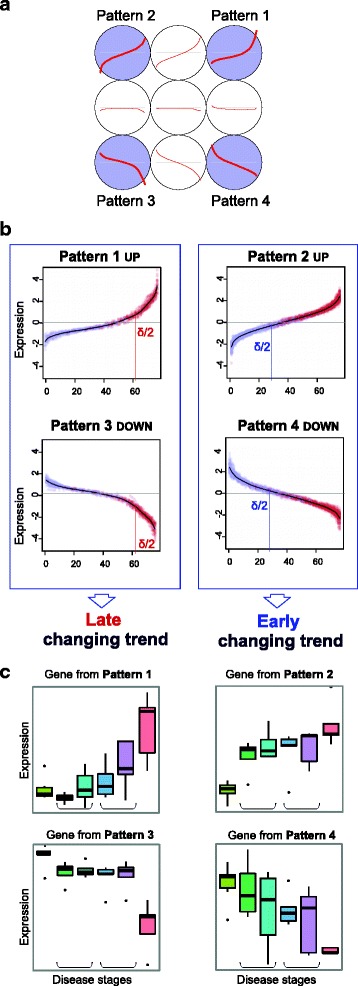
Workflow overview of the results provided by the methodology proposed. **a** Expression patterns (clusters) found using *SOM* on the correlations obtained for each gene along the stages with the *Gamma* rank correlation. Highlighted in blue the 4 patterns selected (for the case of the MDS dataset) as the most representative of 9 profiles explored, which included most of the features and the largest changes: 2 increasing (p1 and p2) and 2 decreasing (p3 and p4). **b** Standardized and sorted expression of the genes included in each pattern. *Blue*: samples in control or initial stages; *red*: samples in late or acute stage; grey: intermediate stages. **c** Boxplots of the expression signals of four example genes that follow each one of the 4 patterns found. These genes also correspond to the MDS dataset and the plots include 6 stages of the disease

In order to find and identify the possible expression patterns within the genes associated to the pathogenesis of the disease, a *Self Organizing Map (SOM)* [17] was applied. *SOM* is a robust method for unsupervised clustering and dimensionality reduction that allows searching for common profiles produced by variables (i.e. by the genes) along a series of conditions (i.e. along multiple samples), grouping such variables according to patterns of similarity found (Fig. 2a). Only the genes that resulted in a significant *Gamma* correlation along the stages of a disease where introduced in the *SOM* analysis. To perform this clustering analysis and pattern recognition, the expression of each gene was standardized by subtracting its mean and dividing by its standard deviation. In this way, the expression of all the genes was within the same scale. This normalization step allowed a better integration of samples from different datasets. For each gene, the standardized expression values were sorted in ascending or descending order (ascending if the mean expression in the last stage was higher than in the first stage, and descending otherwise). This reordering was always done following the expression signal intensity (from high to low, or vice versa) allowing the switch of position of the samples according to their signal. This categorization by intensity did not alter the order of most sample types and it allowed the construction of robust and consistent profiles because it can compensate the effect of some noise points. In fact, in the case of the MDS dataset, we confirmed that most samples in the last stage (AML) were kept on the final positions, the control samples (NoL) on the initial positions and the intermediate-stage samples (MDSs) in the middle (Fig. 2b: sample color). With this normalized and sorted expression data, the genes were clustered with the *SOM* implementation from *Kohonen* R package (version 2.0.19) [18]. In all the analyses performed with the three datasets studied in this work, we used a 3 × 3 grid with rectangular topology, since this allowed allocating up to 9 possible clusters or groups of genes with similar profiles and this was enough in all cases to detect the main patterns (Fig. 2a).

## Results and Discussion

### Patterns found along the progression of diseases: three case studies

In the first dataset studied, corresponding to MDS, we applied the methodology using 2 different ways of grouping the samples: by disease subtypes defined in MDS (6-stage contrast), or by risk of transformation into leukemia (4-stage contrast) (Fig. 1). The final results either way were very similar and coherent with the fact that the 6-stage contrast is a subdivision of the 4-stage contrast. Therefore, most of the genes in the 6-stage contrasts were included in the 4-stage contrasts. This result shows that the method is able to consider different number of levels or stages, instead of being forced to choose *a priori* just one number of stages.

**Fig. 3:**
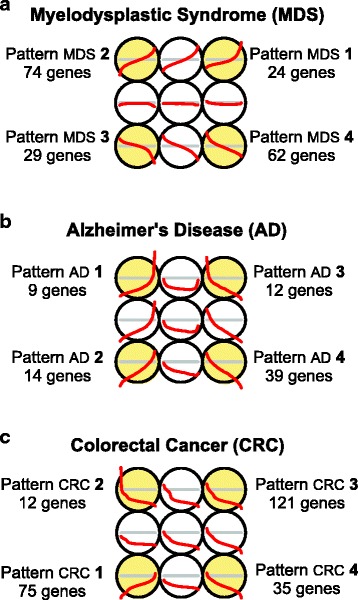
Patterns found in the analyses of the gene-stage expression profiles of 3 disease datasets. **a** Myelodysplastic Syndrome, MDS. **b** Alzheimer’s Disease, AD. **c** Colorectal Cancer, CRC. The results correspond to the outputs of the *SOM* analyses done in all cases with a maximum of 9 (3x3) possible distinct profiles. In all cases 4 significant patterns were found. The number of genes included in each pattern are indicated in each case

The application of the method to the MDS dataset, either considering 4 or 6 stages, revealed the presence of four main patterns, that included most of the genes with a significant correlation (Fig. 2a). Two of the patterns (patterns MDS 2 and 4) had an expression trajectory that follows a quite linear-like slope after a more significant change at the beginning (we named this as *early changing trend*); while the other two patterns (patterns MDS 1 and 3) had a drastic change just at the end, following a linear-to-exponential-like trajectory (we named this as *late changing trend*) (Fig. 2b). The δ/2 threshold within each pattern (i.e. the point that locates the 50 % change in the total range of expression change), allowed to confirm the trends described, because while for patterns MDS 2 and 4 the δ/2 point lies approximately at the initial stages (i.e. at MDSs of low-risk), for patterns MDS 1 and 3, it lies at the end, closer to the acute stage that is AML (Fig. 2b). It is known that myelodysplastic syndromes include a quite heterogeneous set of hematological malignancies. Even within the subgroups currently defined by medical consortiums, there is still considerable clinical heterogeneity [19]. This heterogeneity is also reflected at genetic level, where there are several molecular features known to be associated with MDS (e.g. specific chromosomal alterations or mutations in some specific genes like RUNX1, TP53 or SF3B1), but each of them is not necessarily present in every patient [20, 21]. In this way, the methodology presented provides a powerful alternative to the traditional differential expression analyses, since many of the gene changes that were detected here (Fig. 3a) would be lost with such analyses due to the small significance of the expression changes in pair-wise comparisons between two subtypes and also due to the frequent sample heterogeneity. Comments about the specific genes found and their functions are included below in the last paragraphs of the Results and Discussion section.

The analyses done on the other two datasets of AD and CRC also allowed the discovery of interesting patterns in the progression of these diseases. Figure 3b presents the four main patterns found for Alzheimer (AD) and it can be seen that these trends are steeper than the ones observed for MDS and so they indicate a sharp progression along the analyzed stages. Two of the patterns (patterns AD 1 and 2) correspond to groups of genes with an expression trajectory that increases towards the final stage, i.e. towards severe AD. The other patterns (patterns AD 3 and 4) correspond to groups of genes with trajectory that is high in the control initial samples but decreases sharp towards the final stages of AD. The number of genes grouped in these trends is not very big due to the fact the AD dataset is the one with fewer samples and fewer stages (Table 1). Despite this limitation, the genes found –as discussed below– are quite relevant and show the value of the methodology.

Finally, Fig. 3c presents the four main patterns found for colorectal cancer (CRC) along its progression from early tumor stages to advanced stages. In this case three of the main patterns (patterns CRC 1, 2 and 3) correspond to groups of genes with expression trajectory that decreases towards the final stages of CRC; and only one (i.e. pattern CRC 4) includes genes that have low expression in the control samples that increases in the CRC stages. The patterns found for this dataset also include quite interesting genes associated to CRC, that are discussed below in the last part of this section.

### Patterns found in the simulated RNA-seq dataset

**Fig. 4:**
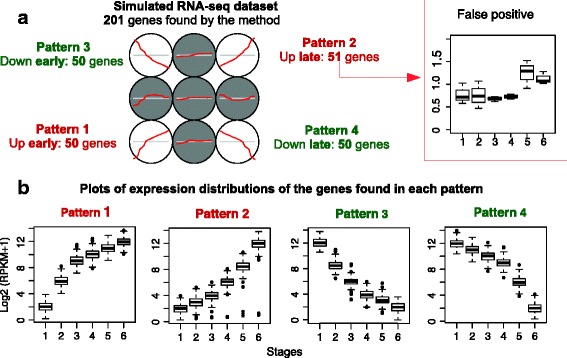
Patterns found in the analyses of the expression patterns of a simultaed RNA-seq dataset. The dataset includes RPKM signals for 1000 genes and 18 samples divided in 6 stages. **a** Outputs of the *SOM* analysis that identifies 4 main patterns including: 50 genes up-early, 51 up-late, 50 down-early and 50 down-late. In this 201 genes found, only one was a false positive and there are not any false negatives. **b** Plots of expression distributions of the genes found in each pattern. The plots represent the expression signal distributions (as log2 of the RPKM values +1) of the genes in each of the 4 patterns

As indicated in the Methods section, we generated a simulated RNA-seq dataset with 1000 genes that models a disease including 18 samples on 6 stages, with a subset of 200 genes that followed 4 patterns of up-regulation or down-regulation along the stages. The application of our methodology on this dataset showed the performance of the method in finding the genes that belong to each pattern. The results are presented in Fig. 4, showing that 201 genes were assigned to 4 patterns, resulting in a very good performance with only 1 false positive included in pattern 2 (i.e., in this pattern the method detected 51 genes instead of 50 genes expected). This analysis indicates a 99 % accuracy and, despite being on simulated data, it shows that the methodology can be applied to different types of data and it is able of finding a signal inside a nonparametric data matrix where 80 % of the genes were not positive. The gene that is “false” had a very low expression level (lower than 1 in most of the samples, see Fig. 4a) and this reveals a common problem in expression profiling where most mistakes are provoked by the low-expression genes, since very small changes on then can mark significant differences.

### Genes associated to the disease progression: functional and biological findings

All the specific genes found on the experimental datasets as associated to the patterns of each disease are included in three Additional files. The genes found for MDS in Additional file 1: Table S1; the genes found for AD in Additional file 2: Table S2; the genes found for CRC in Additional file 3: Table S3. We have also done functional enrichment analyses with the lists of genes associated to some of the patterns found in each one of these diseases, in order to see the biological coherence of the results. These functional enrichment results are included in Additional file 4: Table S4, that presents the enrichment on biological terms associated to the genes included in the following patterns: the expression pattern 1 of MDS (24 genes UP-regulated) (Fig. 3a); the expression pattern 4 of AD (39 genes DOWN-regulated) (Fig. 3b); and the expression patterns 2, 3 and 4 of CRC (168 genes that are all the genes DOWN-regulated in this disease) (Fig. 3c).

The interest of the genes included in the patterns found for each disease is supported by the finding of some genes that are well-known markers of the corresponding disease and the progression of such pathology. This is the case, for example, of the 24 genes included in MDS pattern 1 that are the most clearly UP-regulated genes in the late stages of this disease and, in this way, should mark the entrance in acute leukemia (AML). The enriched functions found show that these genes are related with hematopoiesis (GO:0030097) and mark a negative regulation of myeloid cell differentiation (GO:0045638) indicating a tendency to generate undifferentiated cells (Additional file 4: Table S4A). In fact, many of these genes have been reported as related to AML: ANGPT1, FLT3, HOXA3, HOXA7, HOXA9, HOXB2, HOXB3, MEIS1. For example, FLT3 and MEIS1, are clear AML markers and the appearance up-regulated at the late stages of MDS supports an evolution towards such acute leukemia states [21]. The same occurs with the cluster of HOX genes (HOXA3, HOXA7, HOXA9, HOXB2 and HOXB3) that are well reported as genes that are up-regulated in AML. These results show that the methodology allows finding genes relevant to the analyzed disease.

With respect to new genes found in other MDS patterns, the one assigned to pattern MDS 3 with the most significant *Gamma* correlation was LCN2. Higher LCN2 (lipocalin 2) expression in bone marrow of patients has been recently identified as an independent favorable prognostic factor in acute myeloid leukemia [22]. Therefore, its repression in the progression of MDSs, as we observed when this gene is included in MDS pattern 3, can indicate the propensity towards more malignant states.

With respect to genes marking the early stages of MDS progression, we found that the gene most significantly associated to MDS pattern 4 (showing a *Gamma* correlation of 0.739) was UCA1 (urothelial cancer associated 1), that is a long non-coding RNA (lncRNA). This gene has gained great attention in recent years due to its aberrant expression in a broad range of cancer tissues and cells [23]. We also detected in this pattern ORM1 and ORM2. Human orosomucoid (ORM) is a major acute-phase plasma protein (encoded by 2 highly homologous genes) that is induced as a reaction to inflammation, infection, injury or cancer. In this way it is quite remarkable the observation of the induction of these genes in the MDS pattern 4, that represents an early stage of the disease revealing genes that are up-regulated in the initial low-risk MDS subtypes (as shown with the early changing trend in Fig. 2b).

With regard to AD, despite the fact that the number of samples analyzed for this dataset was smaller than in the other two cases, we found some interesting genes associated to the disease progression patterns. The enrichment analysis done on the genes of AD pattern 4, that is the pattern including most genes (Additional file 4: Table S4B), shows a down-regulation of neuro-transmission and synapsis functions (GO:0007269, GO:0001505, GO:0035249) determined by genes: GLS, GRIA3, LIN7A and SLC17A7. This functional mark would be expected in AD patients in the progression of their disease. By contrast, in the case of AD pattern 1, 9 genes of up-regulation were found and several of these genes have been previously reported as altered in AD, including the top gene of this pattern (with *Gamma* correlation 0.768) that was DEFB125: which is a defensin with expression up-regulated in Alzheimer’s brain [24]. Moreover, gene KIF1B, that was the most significant in AD pattern 2, has been implicated in different forms of human neurodegenerative diseases, playing a role in the function and regulation of synaptic signaling [25]. It is also interesting the identification of gene ARHGAP20 as the most significant included in AD pattern 4, because very recently this gene has been found in the first genome-wide association study (GWAS) of cognitive decline in AD with longitudinal measures of cognition published Sherva et al. [26]. The association of this gene to Alzheimer’s disease severity and progression is a new observation derived from the methodology here proposed, and it is supported by the reported study about the rate of cognitive decline in AD.

Finally, in the case of CRC the enrichment analysis done on the 168 genes that show patterns of down-regulation on this disease (CRC patterns 2, 3 and 4) (Additional file 4: Table S4C), shows multiple functions related to a repression of cellular responses (GO:0071868, GO:0071870, GO:0009611) and negative regulation of cytokine-mediated signaling (GO:0001960) determined by genes: ADIPOQ, CACTIN, GNG2, PDE4D, SLIT3, SNCA. A lack of response to damage on the cells seems to be a signature that these expression patterns reveal for colon cancer progression. Some of the repressed genes (i.e. BCL2, TNFRSF10C and TNFRSF1A) also indicate a negative regulation of apoptosis, that is very much associated with tumor progression. It is also quite interesting to find that one of the most clearly inhibited genes following CRC pattern 2, is SDPR, a cavin family protein (serum deprivation response factor-related gene product) that binds to C-kinase (PKC) and has been found as epigenetically inactivated in gastric cancer and in breast cancer [27]. Removal of this protein causes caveolae loss, i.e. plasma membrane lipid rafts loss, and this membrane vesicle trafficking is an essential function of the normal colon epithelium. Another interesting gene discovered in the repression patterns of CRC (pattern 4) is MTUS1, since it has been recently reported that loss of MTUS1 in gastric cancer promotes tumor growth and metastasis [28]. With respect to CRC pattern 1, that is the only one showing up-regulation, we found reports of some novel genes included in this pattern that can be related to the progression of the disease, like for example squalene epoxidase (SQLE) that is up-regulated in breast cancer and indicates poor clinical outcome in early stages of this disease [29]. Another very interesting protein found in this CRC pattern is KLHL20, a BTB-kelch protein that is involved colon cancer metastasis since it is a substrate adaptor of CUL3 E3 ligase complex catalyzing the ubiquitination of DAPK (death-associated protein kinase) a well-known tumor suppressor [30].

The functional demonstration of the role of each gene included in the patterns found for MDS, AD and CRC on the progression of these diseases is out of the scope of this work; but the results presented in this manuscript provide enough data to shown the biological coherence and consistency of the gene-disease-patterns found and to validate the methodology proposed. In this way, the scope of this work is to present a useful analytical methodology applied to several independent datasets and to provide tools and means to allow that other researchers can use it (supplying an R software to do it). To this scope, we show in this work that several of the genes found for the studied diseases have expected meaning and coherence, and are consisting with multiple published reports. In fact, a deeper biological analysis of MDS (exploring its evolution along stages and the gene patterns that are behind the progression of this disease) will be included in another publication that we are preparing in collaboration with clinical doctors, where we will focused on the biological interpretation of the findings and their relevance to the understanding of the MDS disease. In such complementary work, we have combined more series of MDS produced with different expression platforms (i.e., *Affymetrix* HGU133 plus 2.0 arrays and *Affymetrix* Human Exon arrays). We have also compared the results of these datasets with other independent studies on MDS, like one from pediatric MDS (done on bone marrow mononuclear cells) and another from MDS patients (done on CD34+ cells). All these analyses are not included in the present manuscript, but they allow us to indicate that we have applied the proposed methodology to other datasets of the same disease and we have found similar results.

## Conclusions

The method here presented allows the analysis of the evolution of complex and heterogeneous diseases including different pathological subtypes in stages. The procedure identifies gene groups moving in a coordinated way along a series of associated stages. In particular, the methodology analyses genome-wide expression profiles to find patterns of genes associated to the changes along the progression of the disease stages, showing a new way to achieve a robust profiling of transcriptomic data from different sets of patients that are measured at discrete states along the phases of the disease.

Depending on the scope of each specific study, the focus of the analyses can be on the alterations that happen at initial stages, at the final transformation or even at intermediate stages in a fuzzy way. In order to provide a clearer overview of the studied stages, it is recommended to have at least four or five stages, including two reference stages: a control “initial stage” and a “final stage” corresponding to the most acute state of the disease. As a minimal number of samples required at each stage, we recommend that any study should include at least three biological replicates per stage.

Finally, in this work the methodology has been successfully applied to three independent experimental datasets that study complex diseases. We also applied it to a simulated RNA-seq dataset. The results show that the procedure can be very useful to analyze heterogeneous diseases without the need of having clear subdivisions (i.e., enclosing subtypes that still have to be defined), as long as the samples are placed in stages along the progression of the disease. The results also show that the method is applicable to different types of expression and transcriptomic data, including RNA-seq data.

## Additional files

Additional file 1: Table S1. Significant genes found in the patterns associated to the progression of MDS: A total set of 189 genes were found. These genes are included in one of the 4 patterns identified (marked in red colors in the case of the 2 increasing trends or green colors in the case of the 2 decreasing trends). The *Gamma* correlation factor and the adjusted *p-value* of such correlation are included for each gene in its pattern. (XLSX 79 kb)
Additional file 2: Table S2. Significant genes found in the patterns associated to the progression of AD: In total 74 genes were found as significant assigned to one of the 4 AD patterns. The patterns are marked in red colors in the case of increasing trends or green colors in the case of decreasing trends. The *Gamma* correlation factor and the adjusted *p-value* of such correlation are included for each gene in its pattern. (XLSX 13 kb)
Additional file 3: Table S3. Significant genes found in the patterns associated to the progression of CRC: A total set of 243 genes were included in 4 patterns: 1 increasing and 3 decreasing. Each one of the four patterns is marked in a color (red colors in the case of increasing trends or green colors in the case of decreasing trends). The *Gamma* correlation factor and the adjusted *p-value* of such correlation are included for each gene in its pattern. (XLSX 25 kb)
Additional file 4: Table S4. Functional Enrichment Analyses of lists of genes included in the expression patterns found along the progressive stages of 3 diseases: MDS, AD, CRC. (A) Functional enrichment on terms from Gene Ontology Biological Process (GO-BP) for the 24 genes included in MDS pattern 1. (B) Functional enrichment on GO-BP terms for the 39 genes included in AD pattern 4; (C) Functional enrichment on GO-BP terms for the 168 genes included in CRC patterns 2, 3 and 4. (XLSX 15 kb)

Software script named genediseasePatterns, that allows the use of the method presented in this work using R (software environment for statistical computing: https://www.r-project.org/). This software is provided as a .zip folder (named "genediseasePatterns_R_script.zip") including the following files: 10.1186/s12859-016-1290-4 This software is provided as a .zip folder (named "genediseasePatterns_R_script.zip") including the following files: genediseasePatterns_workflow.html; gene-diseasePatterns_workflow.Rmd; resultGAMMA.RData; resultSOM.RData; GSE28146_series_matrix.txt. The .zip folder is available at: http://bioinfow.dep.usal.es/genediseasePatterns/

## Abbreviations

AD: Alzheimer’s disease
AML: Acute Myeloid Leukemia
CRC: Colorectal cancer
GEO: Gene Expression Omnibus (http://www.ncbi.nlm.nih.gov/geo/)
MDS: Myelodysplastic Syndromes
RAEB: Refractory Anemia with Excess Blasts
RCMD: Refractory Cytopaenia with Multilineage Dysplasia
RCUD: Refractory Cytopaenia with Unilineage Dysplasia
SOM: Self Organizing Map
